# Quantitative SARS-CoV-2 Viral-Load Curves in Paired Saliva Samples and Nasal Swabs Inform Appropriate Respiratory Sampling Site and Analytical Test Sensitivity Required for Earliest Viral Detection

**DOI:** 10.1128/jcm.01785-21

**Published:** 2022-02-16

**Authors:** Emily S. Savela, Alexander Viloria Winnett, Anna E. Romano, Michael K. Porter, Natasha Shelby, Reid Akana, Jenny Ji, Matthew M. Cooper, Noah W. Schlenker, Jessica A. Reyes, Alyssa M. Carter, Jacob T. Barlow, Colten Tognazzini, Matthew Feaster, Ying-Ying Goh, Rustem F. Ismagilov

**Affiliations:** a California Institute of Technology, Pasadena, California, USA; b City of Pasadena Public Health Department, Pasadena, California, USA; University of Iowa College of Medicine

**Keywords:** RT-qPCR, COVID-19, case-ascertained, diagnostics, household study, longitudinal sampling, nasal swab, presymptomatic, saliva, surveillance, transmission

## Abstract

Early detection of SARS-CoV-2 infection is critical to reduce asymptomatic and presymptomatic transmission, curb the spread of variants, and maximize treatment efficacy. Low-analytical-sensitivity nasal-swab testing is commonly used for surveillance and symptomatic testing, but the ability of these tests to detect the earliest stages of infection has not been established. In this study, conducted between September 2020 and June 2021 in the greater Los Angeles County, California, area, initially SARS-CoV-2-negative household contacts of individuals diagnosed with COVID-19 prospectively self-collected paired anterior-nares nasal-swab and saliva samples twice daily for viral-load quantification by high-sensitivity reverse-transcription quantitative PCR (RT-qPCR) and digital-RT-PCR assays. We captured viral-load profiles from the incidence of infection for seven individuals and compared diagnostic sensitivities between respiratory sites. Among unvaccinated persons, testing saliva with a high-analytical-sensitivity assay detected infection up to 4.5 days before viral loads in nasal swabs reached concentrations detectable by low-analytical-sensitivity nasal-swab tests. For most participants, nasal swabs reached higher peak viral loads than saliva but were undetectable or at lower loads during the first few days of infection. High-analytical-sensitivity saliva testing was most reliable for earliest detection. Our study illustrates the value of acquiring early (within hours after a negative high-sensitivity test) viral-load profiles to guide the appropriate analytical sensitivity and respiratory site for detecting earliest infections. Such data are challenging to acquire but critical to designing optimal testing strategies with emerging variants in the current pandemic and to respond to future viral pandemics.

## INTRODUCTION

Early detection of SARS-CoV-2 infection is needed to reduce asymptomatic and presymptomatic transmission, including the introduction and spread of new viral variants. More than half of transmission events occur from presymptomatic or asymptomatic persons (1). Early detection enables individuals to isolate sooner, reducing transmission within households and local communities and to vulnerable populations. Rapid antigen or molecular tests performed on nasal swabs are common for both SARS-CoV-2 screening and symptomatic testing (2) but can have low analytical sensitivity compared with lab-based molecular tests. As new variants of concern emerge with increased transmissibility (3–5), high viral loads (4, 6), and breakthrough infections (7), these testing strategies (analytical sensitivity and sample type) need to be assessed and adjusted to ensure detection of early infection. It is still unclear which testing strategy can detect SARS-CoV-2 infection at the earliest stages. Does one need a high-sensitivity test, or would a test with low analytical sensitivity suffice? Which sample site contains detectable virus first?

Tests with high analytical sensitivity can detect low levels of molecular components of the virus (e.g., RNA or proteins) in a sample. Analytical sensitivity is described by the limit of detection (LOD) of a test (defined as the lowest concentration of the viral molecules that produces 95% or better probability of detection). LOD of SARS-CoV-2 diagnostic tests are described in various units; the most directly comparable among tests are units that report the number of viruses (viral particles) or viral RNA copies per milliliter of sample. Viral RNA copies/mL are roughly equivalent to genome copy equivalents/mL (GCE/mL) or nucleic-acid detectable units/mL (NDU/mL). These LOD values are tabulated by the U.S. Food and Drug Administration (FDA) and vary by ≥5 orders of magnitude between tests (8). Tests with high analytical sensitivity have LOD values equivalent to ∼10^2^ to 10^3^ copies/mL of sample, whereas tests with low analytical sensitivity have LOD values equivalent to ∼10^5^ to 10^7^ copies/mL (9–12). Importantly, test types (e.g., reverse transcription-quantitative PCR [RT-qPCR], antigen) are often incorrectly equated with a certain analytical sensitivity, despite an FDA analysis (8) demonstrating that the sensitivity of different RT-qPCR tests ranges from highly sensitive (e.g., LOD of 180 NDU/mL for PerkinElmer and 450 NDU/mL for Zymo Research) to substantially less sensitive (e.g., LOD of 180,000 NDU/mL for TaqPath COVID-19 combo kit and 540,000 NDU/mL for Lyra Direct SARS-CoV-2 assay). The low end of this range (corresponding to the higher LOD values) overlaps with the range of low-analytical-sensitivity rapid isothermal nucleic acid tests (e.g., LOD of 180,000 NDU/mL for Atila BioSystems and 300,000 NDU/mL for Abbott ID NOW tests) and approaches the analytical sensitivity range of antigen tests (9, 10). To choose the appropriate test for reliable early detection, one needs to measure viral loads present in samples collected early in the course of infection (13) and then choose a test with an LOD below that viral load. Initial data by us (14) and others (15, 16) show that, at least in some humans, SARS-CoV-2 viral load can be low (in the range of 10^3^ to 10^5^ copies per mL of saliva sample) early in infection; therefore, only tests with high analytical sensitivity would reliably detect virus in saliva.

Sampling site or specimen type may also be critical to early detection. Other respiratory viruses have been shown to have detection rates that vary by sampling site (17), which have occasionally been linked to viral tropism. For example, the cellular receptor for entry of Middle East respiratory syndrome coronavirus (MERS-CoV) is expressed nearly exclusively in the lower respiratory tract, prompting recommendations for diagnostic testing of specific sample types (bronchoalveolar lavage fluid, sputum, and tracheal aspirates) (18). A previous study on SARS-CoV found high levels of viral RNA in saliva and throat-wash early in the infection course (before the development of lung lesions), suggesting saliva as a promising sample type for early detection (19). Although nasopharyngeal (NP) swab is often considered the gold standard for SARS-CoV-2 detection, it requires collection by a health care worker and is not well tolerated. Furthermore, the performance of NP swabs for early detection of current SARS-CoV-2 variants is unknown. Sample types such as anterior-nares or midturbinate nasal swabs (20–23) and saliva (24–27) are more practical, especially for repeated sampling in screening.

To understand the required test sensitivity and the optimal sample type for earliest SARS-CoV-2 detection, we designed a case-ascertained study of household transmission with high-frequency sampling of both saliva and anterior-nares nasal swabs. Building on our earlier work (14), we enrolled individuals ages 6 years and older who had recently tested positive (household index case) and their exposed household contacts at risk of infection. Negative samples preceding the first positive result are needed to confirm that a participant is within the first days of detectable SARS-CoV-2 RNA. All participants self-collected saliva and anterior-nares nasal swabs twice daily, in the morning upon waking and before bed. Importantly, all samples were immediately placed in a guanidinium-based inactivating and RNA-stabilizing solution (see Materials and Methods) Samples were screened for SARS-CoV-2 *N1* and *N2* gene positivity using a high-sensitivity assay. When a transmission event was observed (a previously SARS-CoV-2-negative participant tested positive in at least one sample type), we quantified viral loads in all samples prospectively collected from that participant for at least 2 weeks from their first positive result. Quantification was performed via quantitative reverse-transcription PCR (RT-qPCR), with a subset of measurements validated by reverse-transcription droplet digital PCR (RT-ddPCR), capturing the early and full course of acute SARS-CoV-2 infection with high sensitivity.

## MATERIALS AND METHODS

Refer to the supplemental material for detailed methods.

### Questionnaires and sample collection.

Acquisition of participant data was performed as described previously (14). Symptoms (including those listed by the Centers for Disease Control and Prevention [CDC]) were reported by participants twice daily at the time of sample collection (28).

Participants self-collected nasal-swab and saliva samples in the Spectrum SDNA-1000 saliva collection kit (Spectrum Solutions LLC, Draper, UT, USA), which contains 1.5 mL of liquid buffer, at home twice per day (after waking up and before going to bed), per the manufacturer’s guidelines. A parent or legal guardian assisted all minors with collection and were instructed to wear a face covering during supervision.

Samples were stored at 4°C and equilibrated to room temperature before being processed with extraction protocols.

### RNA extraction and nucleic acid quantification.

Participant saliva and anterior-nares swab samples were extracted using the KingFisher Flex 96 instrument (Thermo Fisher Scientific) with the MagMax Viral Pathogen I nucleic acid isolation kit (catalog [cat.] no. A42352; Applied Biosystems, Waltham, MA, USA) guided by Thermo Fisher technical notes for SARS-CoV-2 modification and saliva.

RT-qPCR was performed as previously described (14) using the CDC 2019-novel coronavirus (2019-nCoV) real-time RT-PCR diagnostic panel (29), with duplicate reactions. See the supplemental material methods for establishing the extraction to RT-qPCR assay workflow LOD of 1,000 copies/mL (Fig. S1A and B in the supplemental material).

For samples defined as positive by assay guidelines from the CDC (29), viral load was quantified by conversion of the mean quantification cycle (*C_q_*) of duplicate RT-qPCRs using the equations obtained from calibration curves of contrived samples—healthy human saliva or nasal fluid spiked with heat-inactivated SARS-CoV-2 particles. See the supplemental material methods for additional details.

**FIG 1:**
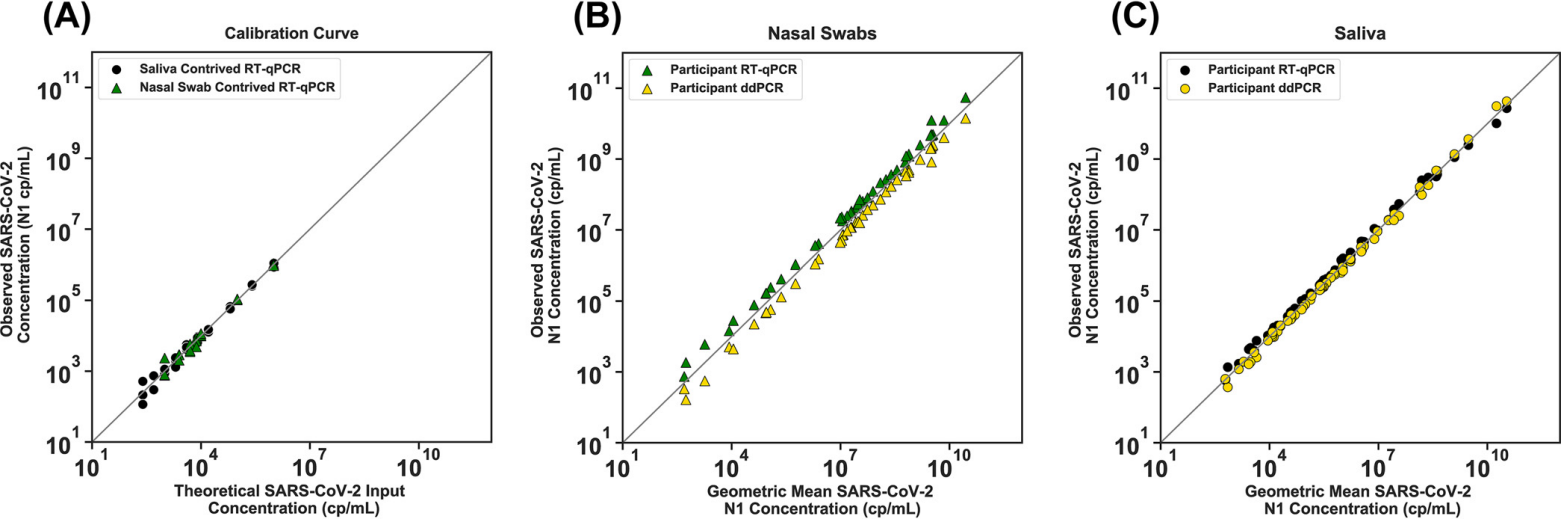
SARS-CoV-2 viral-load quantification measured with RT-ddPCR and RT-qPCR. (A) Calibration curves were prepared with contrived saliva and nasal-swab samples. The theoretical SARS-CoV-2 concentration was calculated from a dilution series of contrived samples that were prepared using commercial, inactivated SARS-CoV-2 and commercially available SARS-CoV-2-negative saliva (black circles) or nasal fluid (green triangles) and run with the CDC SARS-CoV-2 RT-qPCR assay. Detailed calibration curves are shown in Fig. S2. (B) Participant nasal-swab or (C) saliva samples positive for SARS-CoV-2 RNA at a range of viral loads were selected. SARS-CoV-2 *N1* concentrations (copies/mL) by detection method of RT-ddPCR (gold triangles in panel B, gold circles in panel C) and RT-qPCR (green triangles in panel B, black circles in panel C) are plotted against the geometric mean of RT-qPCR and RT-ddPCR viral-load concentrations (the square root of the product of the two viral-load measurements). A total of 42 nasal swab and 63 saliva samples from study participants were quantified with both methods. The gray line represents *x* = *y*. See the supplemental material methods for details of contrived samples, calibration curves, and calculations.

Quantification was also performed by reverse-transcription droplet digital PCR (RT-ddPCR) on both the calibration curve samples (Fig. 1, Fig. S2) and participant samples (Fig. 1) using the Bio-Rad SARS-CoV-2 droplet digital PCR kit (cat. no. 12013743; Bio-Rad). Droplets were created using the QX200 droplet generator (cat. no. 1864002; Bio-Rad); thermocycling was performed on a Bio-Rad C1000 and detected using the QX200 droplet digital PCR system (cat. no. 1864001; Bio-Rad). Samples were analyzed with QuantaSoft analysis Pro 1.0.595 software following Bio-Rad’s research-use only (RUO) SARS-CoV-2 guidelines (30).

### Viral sequencing.

Saliva and nasal-swab samples with an *N1* gene *C_q_* of below 26 were sent to Chan Zuckerberg Biohub for SARS-CoV-2 viral genome sequencing, a modification of Deng et al. (31) as described in Gorzynski et al. (32). Sequences were assigned pangolin lineages described by Rambaut et al. (33) using Phylogenetic Assignment of Named Global outbreak LINeages software 2.3.2 (github.com/cov-lineages/pangolin). Chan Zuckerberg Biohub submitted the resulting genomes to GISAID.

**Data availability.** Data are available on CaltechDATA at https://data.caltech.edu/records/1942.

## RESULTS

We first established and validated two independent quantitative assays to measure SARS-CoV-2 viral load, an RT-qPCR based on the assay put forth by the U.S. Centers for Disease Control and Prevention (CDC) (29) and an RT-ddPCR assay developed by Bio-Rad (30). Both of these assays received an emergency use authorization (EUA) for qualitative, but not quantitative, detection of SARS-CoV-2. We optimized the extraction and each quantitative assay protocol (see supplemental material methods) to obtain more reliable quantification of SARS-CoV-2 viral load. The LOD of the modified assay was determined to be 1,000 copies/mL or better by following FDA guidelines (see Materials and Methods; Fig. S1). Commercial, heat-inactivated SARS-CoV-2 virus was used to establish calibration curves to convert RT-qPCR quantification cycle values (*C_q_*) to viral load (Fig. 1A; full details in Fig. S2 and supplemental material methods). The linearity of these calibration curves was assessed with 43 participant nasal swab (Fig. 1B) and 63 participant saliva samples (Fig. 1C) across a wide dynamic range of viral loads.

**FIG 2:**
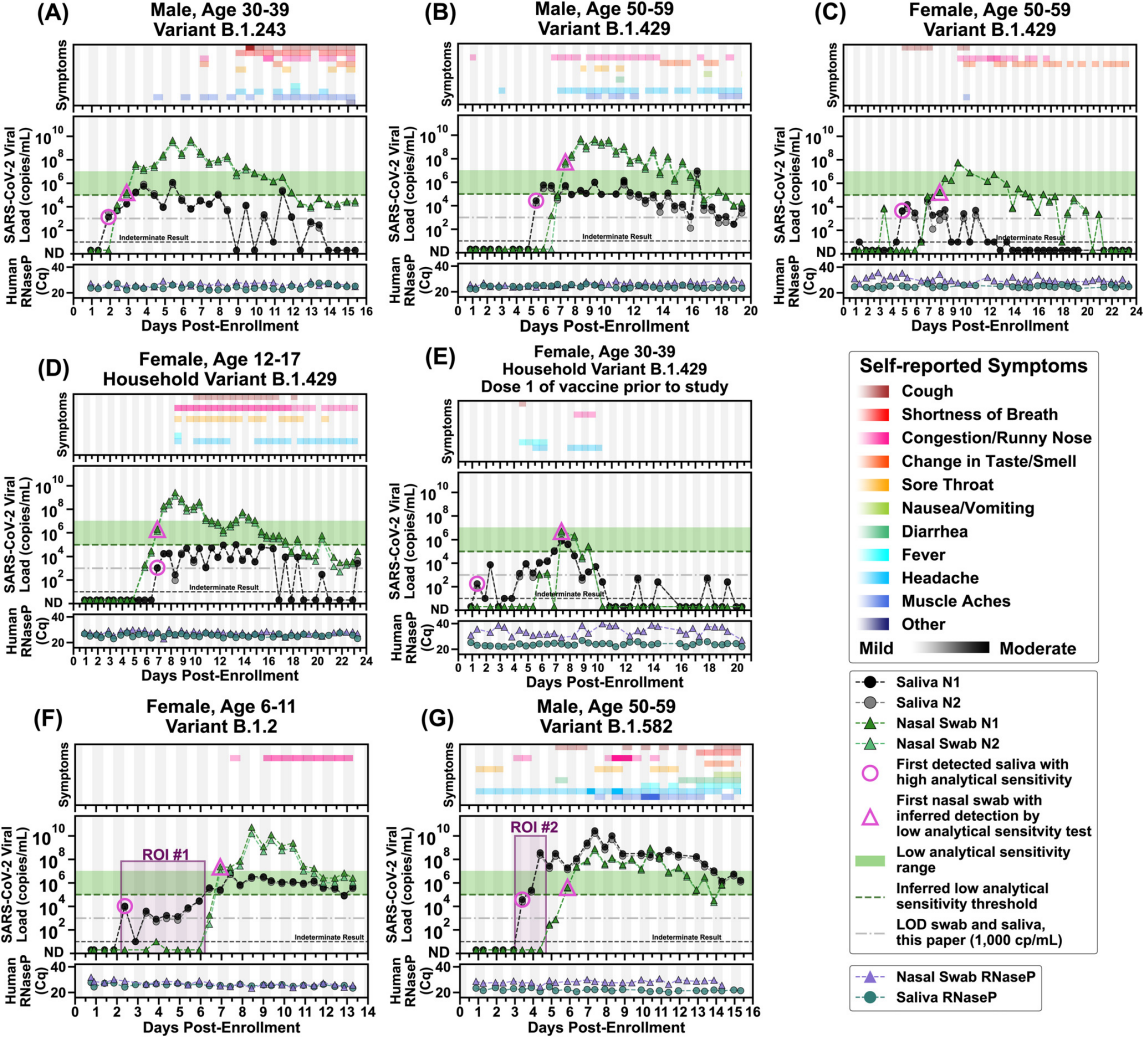
Symptoms and SARS-CoV-2 viral loads in paired saliva and nasal-swab samples of seven participants who became SARS-CoV-2 positive during study participation. (A to G) Self-reported twice-daily symptom data over the course of enrollment are shown as the top panel for each of the participants (see color-coded legend for symptom categories). Details of symptoms are included in the raw data files. Demographic data including any reported medical conditions are included in Table S1. Viral loads are reported for the *N1* and *N2* genes of SARS-CoV-2 for both saliva (black and gray circles) and nasal-swab samples (dark-green and light-green triangles); ND, not detected for *C_q_*s of ≥40. Samples with an indeterminate result by the CDC RT-qPCR assay are shown along the horizontal black dashed line (see Materials and Methods for details). The limit of detection (LOD) of the assay used for high-analytical-sensitivity measurements is shown with a horizontal gray dashed line. The inferred low-analytical-sensitivity threshold (1.0 × 10^5^ copies/mL) is indicated by the horizontal green dashed line; the low-analytical-sensitivity range (horizontal green bar) is shown. A diagnostic test does not provide reliable detection for samples with viral loads below its LOD. For each participant, the first detected saliva point is emphasized with a pink circle (high analytical sensitivity), and the first nasal-swab point with a viral-load concentration at or above 1.0 × 10^5^ copies/mL (low-analytical-sensitivity threshold) is emphasized with a pink triangle. Vertical shading in gray indicates nighttime (8 pm to 8 am). Internal controls of *RNase P* gene *C_q_*s from the CDC primer set are provided for each sample to compare self-sampling consistency and sample integrity (failed samples, where *RNase* *P C_q_* is ≥40, are not plotted). Participant sex, age range, and SARS-CoV-2 variant are given in each panel’s title. Two regions of interest (ROI) are indicated by purple-shaded rectangles and discussed in the main text.

Next, to quantify the viral load at the earliest stage of infection, we analyzed the viral loads in the saliva and nasal swabs of participants who were negative in both sample types upon enrollment and became positive during their participation in the study (Fig. 2). We extended each participant’s enrollment in our study to acquire 14 days of paired saliva and nasal-swab samples starting from the first positive sample. The data in Fig. 2 report the viral-load concentrations as measured on the day of extraction. All samples were stored at 4°C before extraction; time of storage varied between 0 and 27 days. The stability of SARS-CoV-2 RNA and impact on our conclusions is discussed in the supplemental material methods, Fig. S3, and Fig. S4.

Here, we report complete viral-load curves in saliva and anterior-nares nasal swabs from seven individuals (Fig. 2). Each participant tested negative (ND, not detected; Fig. 2) in both saliva and nasal swabs upon study enrollment, demonstrating that we captured the earliest days of infection. *RNase* *P C_q_* values remained consistent throughout the collection period for saliva and swabs for most participants (Fig. 2A, B, D, F, and G), indicating that observed changes in viral loads were likely not a sampling artifact but reflected the underlying biology of the infection. Because nasal swabs are commonly used with tests of low analytical sensitivity, and because such tests are proposed to be utilized for SARS-CoV-2 serial screening testing (34, 35), we wanted to compare whether low-analytical-sensitivity testing with nasal swabs could provide equivalent performance to high-analytical-sensitivity testing with saliva (26, 36, 37). We did not run any tests with low analytical sensitivity; our quantitative viral-load measurements were used to infer the performance of a test with an LOD representing low analytical sensitivity. When viral loads in nasal swabs crossed a threshold of 1.0 × 10^5^ copies/mL, entering the low-analytical-sensitivity range, shown as the inferred low-analytical-sensitivity threshold (Fig. 2), we marked the sample with a pink triangle.

In six out of seven participants, high-analytical-sensitivity saliva testing would have been superior for early detection of SARS-CoV-2 infection compared with the predicted performance of nasal-swab tests with low analytical sensitivity. This prediction was made by evaluating when nasal-swab viral loads entered the LOD range of nasal-swab tests with low analytical sensitivity. In the seventh participant, the first positive high-analytical-sensitivity saliva test was detected at the same time point that the first nasal-swab test reached a viral load likely to be detected by a low-analytical-sensitivity nasal-swab test (Fig. 2D). In the first participant (Fig. 2A), detection occurred first in saliva at low viral load (1.3 × 10^3^ copies/mL *N1* gene, pink circle), while the nasal swab remained negative, and days before the participant reported any symptoms. As measured, viral load in nasal-swab samples reached the level of LOD of low-analytical-sensitivity tests 1.0 days after the first saliva positive samples (pink triangle). This same pattern of earlier detection in high-sensitivity saliva was observed in five of the other six participants; high-sensitivity saliva was 2.5 days earlier (Fig. 2B), 3.0 days earlier (Fig. 2C), 6.0 days earlier (Fig. 2E), 4.5 days earlier (Fig. 2F), and 2.5 days earlier (Fig. 2G). The maximum delay in detection between saliva and nasal swab in an unvaccinated person was observed in the youngest participant in our study (see region of interest [ROI] no. 1 of Fig. 2F). This participant had detectable but low viral load (10^3^ to 10^4^) in saliva for 4 days, while nasal swabs remained negative by high-sensitivity measurements. Nasal viral loads spiked above 10^10^ copies/mL while the participant’s only symptoms were mild congestion/runny nose.

Even with high-analytical-sensitivity nasal-swab testing, only one participant tested positive in nasal swab before saliva (Fig. 2D). In this participant, SARS-CoV-2 RNA was detectable with a high-analytical-sensitivity nasal swab 1 day before it was detectable in a high-analytical-sensitivity saliva test. Nasal swabs reached the detection range of low-analytical-sensitivity tests (pink triangle) on the same day as the first saliva sample was detected by high-analytical sensitivity testing (pink circle). For all seven participants, high-analytical-sensitivity saliva testing would have detected SARS-CoV-2 RNA either the same day or up to 6 days before viral loads in nasal swab reached the detection limits of low-sensitivity nasal-swab tests.

Two participants (Fig. 2C and E) had low viral load in both saliva and nasal swabs. Their viral-load measurements were near the LOD of our assay, and therefore, as expected, many samples from these participants had indeterminate results. One participant (Fig. 2E) had received one dose of the Pfizer-BioNTech COVID-19 vaccine (38) 13 days prior to her first sample, though observations here are not powered to make conclusions about viral load due to vaccination.

Remarkably (see ROI no. 2 in Fig. 2G), in one participant, saliva viral load spiked to 3.7 × 10^8^ viral copies/mL (*N1* gene target) while SARS-CoV-2 RNA remained undetectable in nasal swab, even by the high-analytical-sensitivity assay used here.

**FIG 3:**
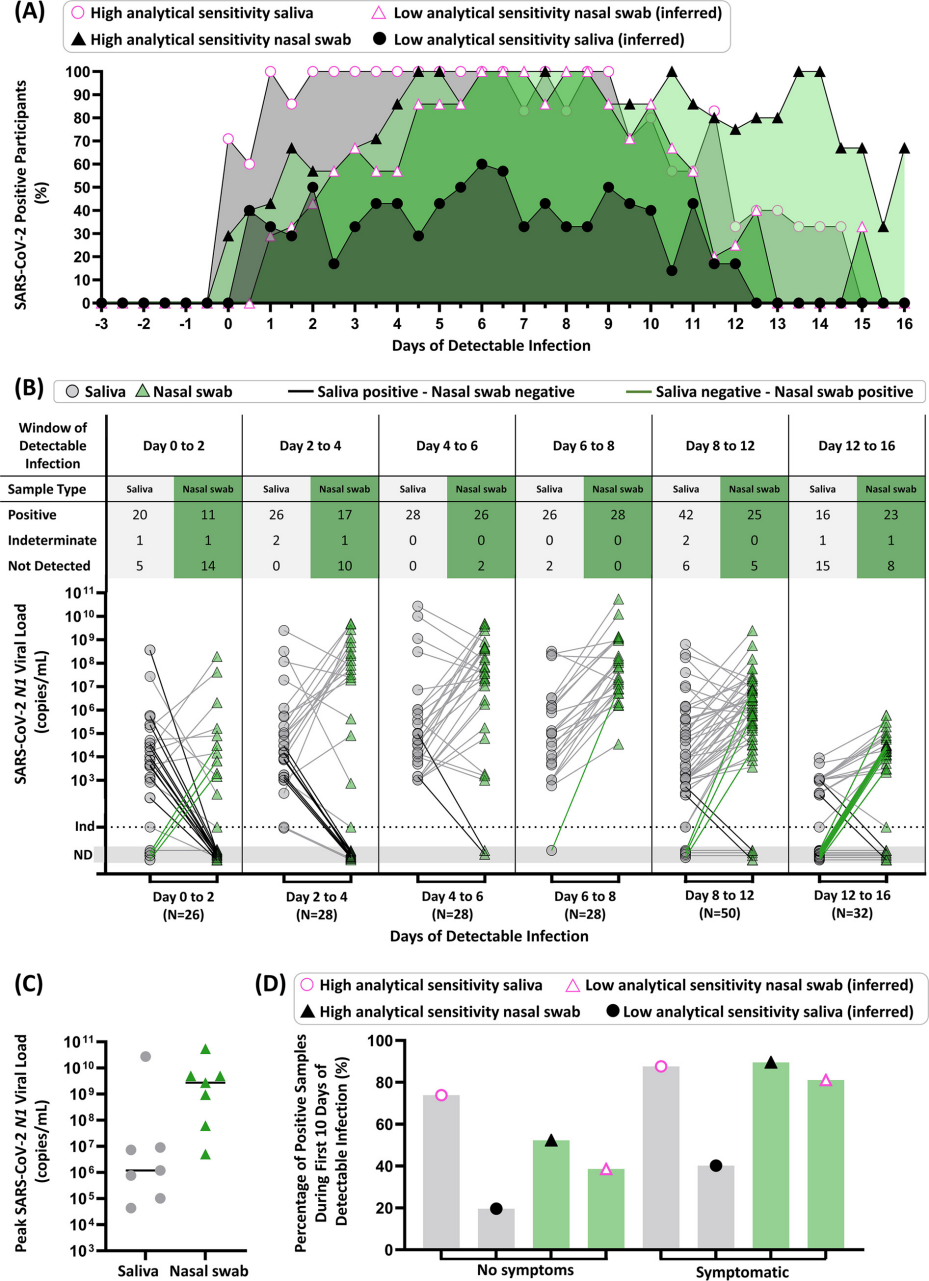
Summary of diagnostic insights from study participants who became infected with SARS-CoV-2 while enrolled in the study. (A) Participant infection time courses were aligned to the first high-sensitivity (LOD of ≤1 × 10^3^ copies/mL) positive result from either saliva or nasal-swab sample type (day 0), and the percentage of positive tests was calculated for each time point (0.5-day intervals) from the first positive sample. The predicted performance of nasal swabs or saliva with low analytical sensitivity was determined using the SARS-CoV-2 *N1* viral-load values for each participant shown in Fig. 2, above a viral-load threshold of 1.0 × 10^5^ copies/mL, entering the low-analytical-sensitivity range. We show the percentage of participants who were detected by our high-analytical-sensitivity saliva test (pink circle), high-analytical-sensitivity nasal-swab test (black triangle), or that could be inferred to be detectable by a low-analytical-sensitivity test nasal-swab (pink triangle) or saliva test (black circle) at a given time point. (B) Quantitative SARS-CoV-2 *N1* viral loads of paired samples collected during time windows of the infection (aligned to first positive result by high-sensitivity testing of either sample type) are shown for saliva (gray circles) and nasal swabs (green triangles). Paired samples for a given time point are connected with gray lines, with emphasis on paired samples where only saliva (black connecting line) or nasal swab (green connecting line) was positive. ND, not detected; Ind, indeterminate result. (C) Peak SARS-CoV-2 *N1* viral loads measured in saliva (gray circles) and nasal swab (green triangles) for each of the seven participants are shown. Horizontal black line indicates the median. (D) Percentage of positive samples (out of all samples of that type and symptom status) are shown for the first 10 days of detectable infection for each participant. Saliva (gray bars with circles) and nasal swab (green bars with triangles) are shown. Positivity by a high-analytical-sensitivity test was observed by our assay, while positivity by a low-analytical-sensitivity test was inferred if the sample had a viral load above 1.0 × 10^5^ copies/mL. The symptom status was classified as symptomatic if the participant reported one or more COVID-19-like symptoms at the time of sample collection. Details of the data analysis are included in the supplemental material methods.

Compiled data from all seven participants highlight the importance of the interplay among anatomical sampling site, infection stage, and diagnostic test sensitivity (Fig. 3). Participant results were aligned to the first positive result from either sample type (day 0). If a saliva or nasal-swab sample had a SARS-CoV-2 viral load above 1.0 × 10^5^ copies/mL, entering the low-analytical-sensitivity range (39), we inferred that a low-analytical-sensitivity test would have correctly determined that sample to be positive. The percentage of participants with either observed or inferred positive results at each time point (0.5-day intervals) from the first positive sample revealed that high-analytical-sensitivity saliva testing outperformed low-analytical-sensitivity nasal-swab testing for the first 5.5 days of detectable infection (Fig. 3A) and high-analytical-sensitivity nasal-swab testing during the first 4 days (Fig. 3A). Analytical sensitivity affects the overall test performance in each sample type. Based on early viral loads in saliva, we inferred that low-sensitivity saliva testing was outperformed by high-sensitivity saliva and both high- and low-sensitivity nasal-swab testing (Fig. 3A).

Next, we plotted paired viral loads in each respiratory site starting from the first positive test (Fig. 3B). From day 0 to day 6, using high-sensitivity testing for both sample types, saliva was more frequently positive than nasal swabs (Fig. 3B). Comparison of paired samples between day 6 and day 12 for both sample types showed highly concordant detection. In a later time interval, between days 12 and 16, nasal swabs were more frequently positive than saliva (Fig. 3B). The median of peak viral loads was higher in nasal swabs than saliva (Fig. 3C), consistent with the literature (21, 23, 40).

Many testing strategies and decisions are based on the presence or absence of symptoms (2, 41). We considered the positivity rate of high- or low-analytical-sensitivity testing methods with each sample type during the first 10 days of test-positive infection (to capture the presymptomatic and symptomatic phases of infection for this cohort, not the postsymptomatic phase), separating them into categories of no symptoms or symptomatic if the participant reported at least one COVID-19-like symptom (Fig. 3D). For samples collected while participants were asymptomatic, high-sensitivity saliva testing was more effective (74% positivity) than high- (52%) or low-sensitivity (39%) nasal-swab testing and low-sensitivity saliva testing (20%). In contrast, during symptomatic phases, which are often concurrent with peak nasal viral loads (Fig. 2), high-sensitivity saliva (88%) and high-sensitivity nasal-swab testing (89%) have similar positivity rates (Fig. 3D). Additionally, based on our measured viral loads, low-sensitivity nasal-swab testing is predicted to perform better in symptomatic cohorts (81%) than in asymptomatic persons (39%), consistent with how these tests were originally authorized.

These data reveal a more nuanced view than “saliva is better than swab.” Using tests with high analytical sensitivity, SARS-CoV-2 RNA is more detectable in saliva than nasal swab during the early phase of the infection (Fig. 3B). However, because viral loads in saliva generally remained lower than those in nasal swabs (Fig. 3C), we infer that positivity by a low-analytical-sensitivity saliva test would be outperformed by both high- and low-analytical-sensitivity nasal-swab testing (Fig. 3A), independent of symptom status (Fig. 3D). It was the combination of test analytical sensitivity along with sample type that determined the overall test performance.

## DISCUSSION

**Limitations.** Our study needs to be interpreted in the context of its limitations. First, our results capture viral-load dynamics from a limited number of individuals from one region of one country with limited SARS-CoV-2 diversity. Follow-up studies with a larger sample size, including individuals of diverse ages, genetic backgrounds, medical conditions, COVID-19 severity, and SARS-CoV-2 lineages would be ideal to provide a more nuanced and representative understanding of viral dynamics in saliva and nasal-swab samples. Second, the commercial inactivating buffer used here (Spectrum SDNA-1000) is not authorized (at the time of this writing) for the sample collection of nasal swabs. The solution in the Spectrum SDNA-1000 kits is a guanidinium-based inactivating and stabilizing buffer that preserves viral RNA but eliminated the opportunity to also perform viral culture. Third, we have paired data for saliva and anterior-nares nasal swabs but do not compare nasopharyngeal (NP) swabs, sputum, or other lower-respiratory specimens. We do not know whether other sampling sites, such as NP swabs or oropharyngeal swabs, would have provided earlier or later detection than saliva. Fourth, we are inferring the ability of tests with low analytical sensitivity to detect infections based on the quantified viral load in the participant samples and the LODs reported by the FDA for the diagnostic platforms. Fifth, some degradation may have occurred in some samples (see supplemental material for a complete analysis of RNA stability). Sixth, all samples were self-collected, which may result in lower-quality specimens.

**Conclusions.** By rapidly enrolling household members at high risk for contracting COVID-19 and having them self-sample twice daily in paired respiratory sites, we observed patterns in SARS-CoV-2 viral load in the earliest days of infection. All seven participants tested negative in saliva and nasal swabs upon enrollment, demonstrating that we captured the earliest detectable SARS-CoV-2 viral load (within 12 h) in both sample types. Our data set helps inform diagnostic testing strategies by showing differences in viral loads in paired nasal swabs and saliva samples at high temporal resolution during the early days and presymptomatic phases of infection.

We made five conclusions from our study.

First, choosing the correct respiratory sampling site is critical for earliest detection of SARS-CoV-2 infection. In our study, alignment of longitudinal data to the first day of positivity clearly shows the superiority of high-sensitivity saliva testing for detection in the first 5.5 days of infection (Fig. 3A). Given our data, early infection viral-load dynamics in multiple sampling sites should be investigated and compared with saliva as new SARS-CoV-2 variants emerge.

Second, our data explain the conflicting results in the literature comparing test performance in paired respiratory sites, with some studies showing that nasal swabs outperform saliva (21, 23, 40) and others showing that saliva (or oral fluid) has detection equivalent to or better than that of nasal swabs (16, 25, 42–50). Through longitudinal rather than cross-sectional sampling, we show that the relative viral loads in each respiratory site are a factor of infection stage (shown in time intervals in Fig. 3B), and the kinetics of viral load may be quite distinct in each sample type for an individual (Fig. 2). Most studies examining paired sample types enrolled participants after a positive test or symptom onset; as our data show, detectable viral loads precede symptoms, in most cases (5 of 7 participants) by several days (Fig. 2).

Third, peak viral load measured in nasal swabs (Fig. 3C) is not representative of detectable viral load in the earliest days of infection (Fig. 3A) or during the presymptomatic phase (Fig. 3D). Early in an infection, it is inappropriate to assume that a person is “not infectious” or “has low viral load” based on a measurement from a single sample type, such as a nasal swab, given that saliva is known to carry infectious virus (51). In our study, we observed a participant with very high (>10^7^ to 10^8^ copies/mL) viral load in saliva samples while the paired nasal swab was either negative (Fig. 2G, ROI no. 2) or had low (∼10^3^ copies/mL) viral load (Fig. 2G, day after ROI no. 2). Quantitative SARS-CoV-2 culture from paired saliva and swab samples is still needed to understand infectiousness during the early stages of SARS-CoV-2 infection.

Fourth, using a diagnostic test with high analytical sensitivity (Fig. 3D), rather than a test of a particular detection method (RT-qPCR, antigen, next-generation sequencing, etc.), is essential to early detection. With many strategies for asymptomatic screening/surveillance testing in use, it is critically important to consider whether the LODs of the tests would be able to detect early infection and to prompt actions that minimize transmission.

Fifth, our data show the utility of combining knowledge of the appropriate respiratory site and the appropriate test analytical sensitivity for achieving earliest detection. Among our unvaccinated participants, when a high-sensitivity test was combined with saliva as a sample type, SARS-CoV-2 infection was detected up to 4.5 days before viral loads in nasal swabs reached the LODs of low-analytical-sensitivity tests (Fig. 2F). Although high-sensitivity saliva testing was usually able to detect virus earlier than nasal swabs (Fig. 3A), during the peak of the infection viral loads in nasal swabs were usually higher than in saliva (Fig. 3C). Furthermore, SARS-CoV-2 was detected in saliva with high-sensitivity methods, and the viral loads were low (Fig. 2, 3A and D); low-sensitivity saliva tests would likely not have been able to detect these infections early. These observations support the preferred use of nasal swabs in environments where only low-sensitivity testing is available, although the performance of such testing for early detection is poor (Fig. 3D). These observations also show that the optimal respiratory sampling site is nuanced and depends on the phase of the infection being detected (early versus peak) and on the analytical sensitivity of the test being used with each sample type.

Our work suggests four steps to improve the effectiveness of diagnostic tests in early detection and preventing transmission of SARS-CoV-2 as new variants emerge and as infections spread to additional segments of the global population. (i) Additional longitudinal studies are needed that include high-frequency collection from multiple respiratory sites using quantitative assays with high analytical sensitivity. (ii) Policy makers need to use such quantitative data to revise and optimize screening testing guidelines to ensure early detection of SARS-CoV-2 infections and reduction of transmission. (iii) Innovation is needed to produce rapid point-of-care tests with high analytical sensitivity for a range of sample types (including saliva) at a price point to enable global distribution. (iv) Quantitative studies of the kinetics of early-stage viral loads in each respiratory site (collected in parallel with viral culture data) must be updated with the emergence of new SARS-CoV-2 variants.

We hope our data, important work by others in this area (15, 16, 51, 52), and future quantitative studies of early viral-load kinetics will lead to improved testing strategies to combat the current COVID-19 pandemic. The methodology for performing such studies efficiently and quickly will likely be extendable to defining strategies for early detection of causative pathogens in subsequent pandemics.
