# Supplementary material for: Quantitative SARS-CoV-2 Viral-Load Curves in Paired Saliva Samples and Nasal Swabs Inform Appropriate Respiratory Sampling Site and Analytical Test Sensitivity Required for Earliest Viral Detection
Source: J Clin Microbiol. 2022 Feb 16;60(2):e01785-21. doi: 10.1128/jcm.01785-21 (PMC8849374; doi:10.1128/jcm.01785-21)
Supplement: Supplemental file 1 — Fig. S1 to S4, Table S1, and supplemental methods and discussion. Download jcm.01785-21-s0001.pdf, PDF file, 1.6 MB [file jcm.01785-21-s0001.pdf]

Supplemental Information for:

Quantitative SARS-CoV-2 viral-load curves in paired saliva and nasal swabs inform appropriate respiratory sampling site and analytical test sensitivity required for earliest viral detection

Emily S. Savela[1#], Alexander Viloria Winnett[1#], Anna E. Romano[1#], Michael K. Porter[1], Natasha Shelby[1], Reid Akana[1], Jenny Ji[1], Matthew M. Cooper[1], Noah W. Schlenker[1], Jessica A. Reyes[1], Alyssa M. Carter[1], Jacob T. Barlow[1], Colten Tognazzini[2], Matthew Feaster[2], Ying-Ying Goh[2], Rustem F. Ismagilov[1]*

[1]California Institute of Technology, 1200 E. California Blvd., Pasadena, CA, USA 91125
[2]City of Pasadena Public Health Department, 1845 N. Fair Oaks Ave., Pasadena, CA, USA 91103

*Correspondence to: rustem.admin@caltech.edu
[#] these authors contributed equally

Contents

- Figures S1 – S4
- Table S1
- Supplementary Methods
- Supplementary Discussion
- Supplementary References
- Author Contributions

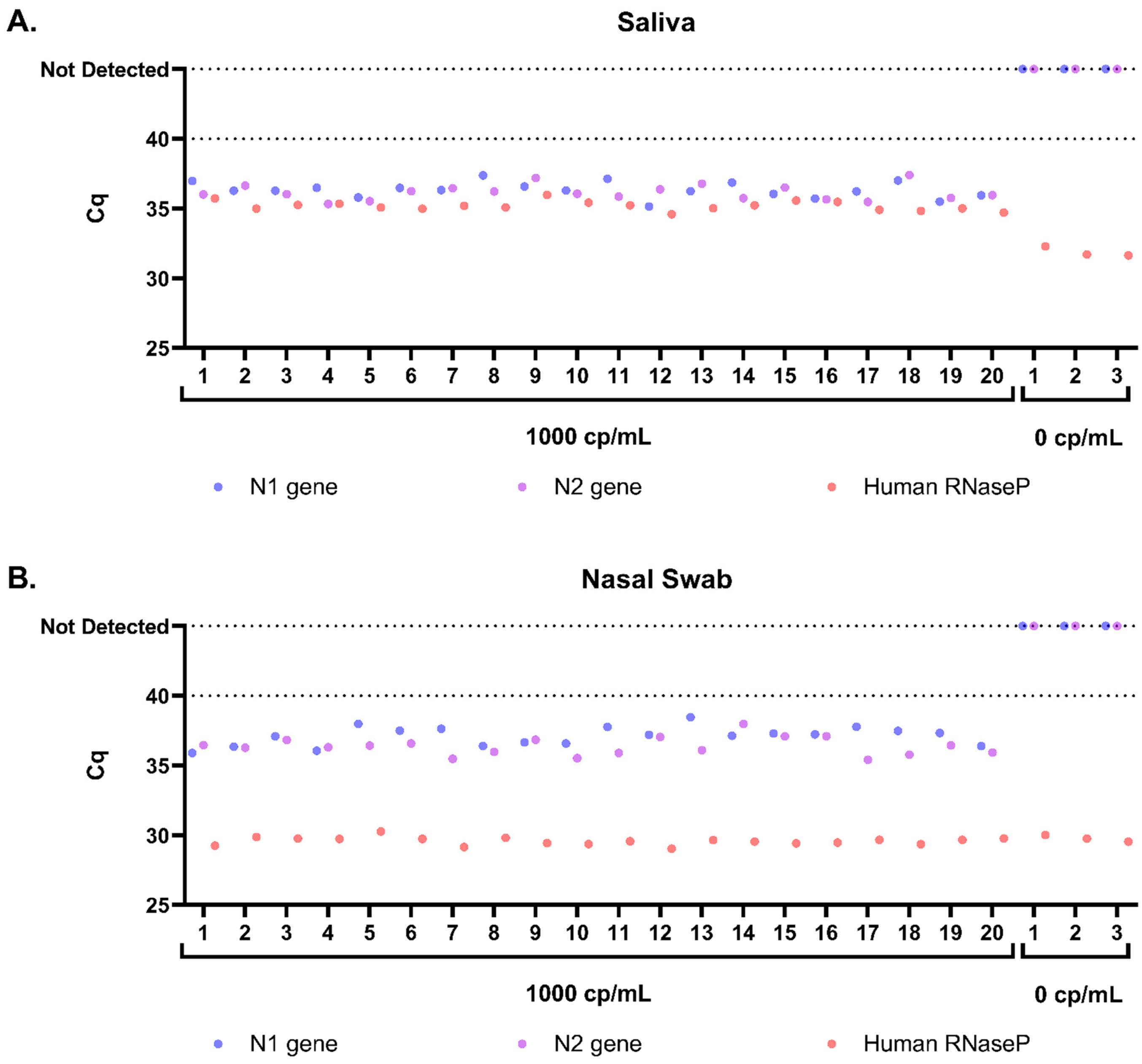

Figure S1. Limit of detection of saliva and nasal-swab RT-qPCR assays used in this study. RT-qPCR quantification cycle (Cq) for SARS-CoV-2 *N1* gene (blue circle), *N2* gene (purple circle), and human *RNase P* gene (orange circle) in 20 replicates of pooled matrix spiked with 1000 copies/mL (cp/mL) heat-inactivated SARS-CoV-2 RNA and 3 replicates of pooled matrix spiked with a buffer blank for saliva (A) and nasal-swab (B) samples. Duplicate RT-qPCR reactions were performed for each extraction replicate and the averages are shown, with the following three exceptions: replicate 9 (saliva), in which the *N1* gene only amplified in 1 of the duplicate runs (*N2* in this run was positive, so per CDC EUA guidelines[1] this run was interpreted as inconclusive), replicate 10 (nasal swab) in which the *N2* gene only amplified in 1 of the duplicate runs (N1 in this run was positive, so this run was interpreted as inconclusive), and replicate 18 (nasal swab) in which the N1 gene only amplified in 1 of the duplicate runs (*N2* in this run was positive, so this run was also interpreted as inconclusive). None of the samples spiked at 1000 copies/mL gave a negative detection result.

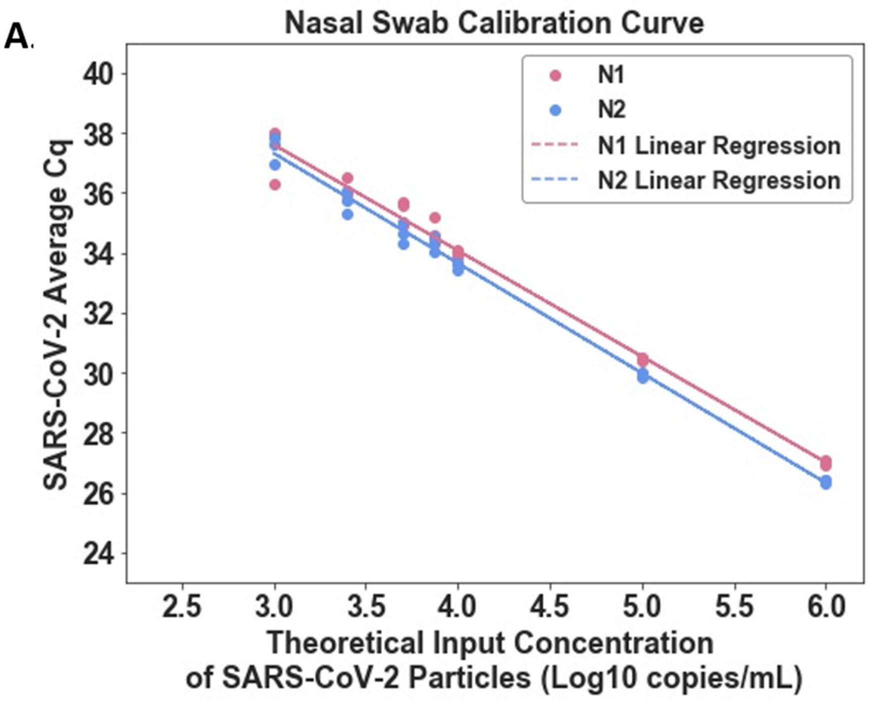

Figure S2. Calibration curve of SARS-CoV-2 inactivated particles to establish viral-load conversion equations. Linear regression of RT-qPCR quantification cycle (Cq) for *N1* (red circle) and *N2* (blue circles) genes at known concentrations of inactivated SARS-CoV-2 particles for saliva (A) or nasal swab (B) using this study's collection and laboratory workflows. Triplicate replicates per concentration were performed. Linear regression for *N1* represented by red line and *N2* represented by blue line. Linear regression R^2 values are 0.986 for *N1* in nasal swabs, 0.994 for *N2* in nasal swabs, 0.989 for *N1* in saliva, and 0.979 for *N2* in saliva.

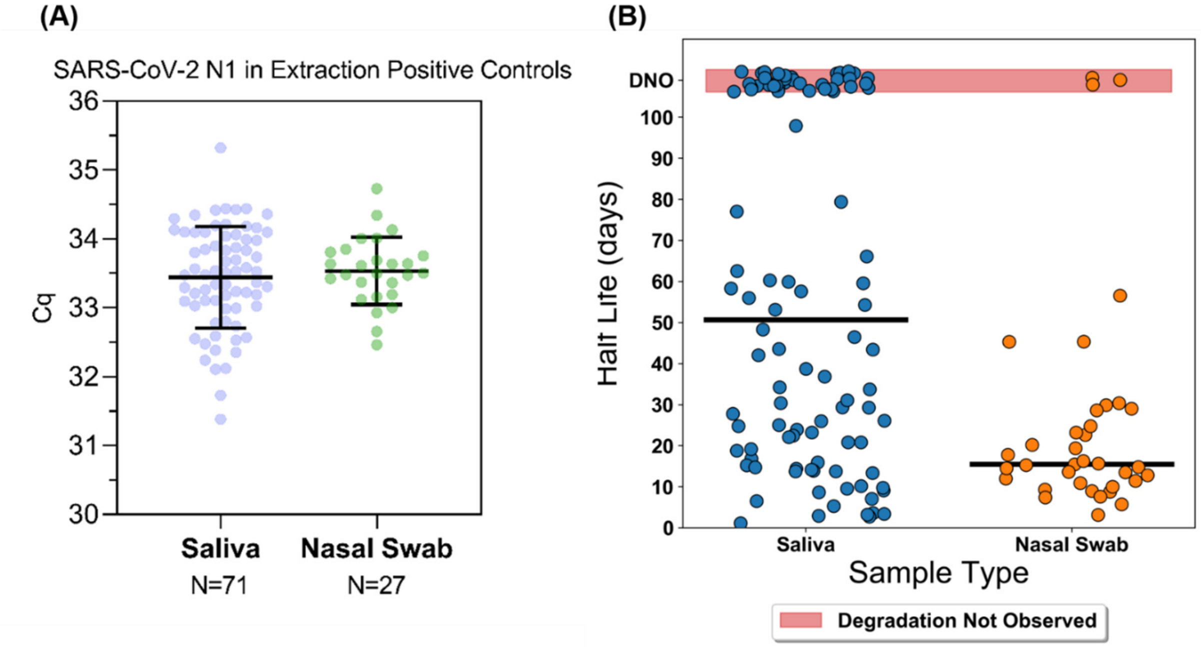

Figure S3. SARS-CoV-2 RNA stability over time in Spectrum SDNA-1000 buffer at 4 °C. (A) Positive extraction control samples from 71 saliva extraction runs and 27 nasal-swab extraction runs are included to show the measurement noise in the quantification workflow. The standard deviation for the positive control measurements was 0.74 Cq for saliva and 0.49 for nasal swab. (B) The observed half-life (days) of participant saliva (blue circles) and nasal-swab (orange circles) samples in Spectrum SDNA-1000 buffer stored at 4 °C. Individual samples were extracted at multiple time points. Half-life in this context refers to the time required to observe a 1 Cq increase (representing a 2-fold decrease) in RNA detected by RT-qPCR. The median point is identified for each sample type (black bars), at 15.0 days for nasal swabs (red circle) and 51.0 days for saliva (green circle). Of the 110 total participant saliva samples plotted in panel B, 36 samples had no evidence of degradation (DNO) under the time frame measured. Only 3 of the 36 total participant nasal-swab samples plotted in panel B had no evidence of degradation under the timeframe measured. DNO = degradation not observed, meaning that the difference in extraction Cq values of the same sample at multiple time points was within 1 standard deviation observed in replicate extraction positive controls for the respective sample type, as shown in panel A.

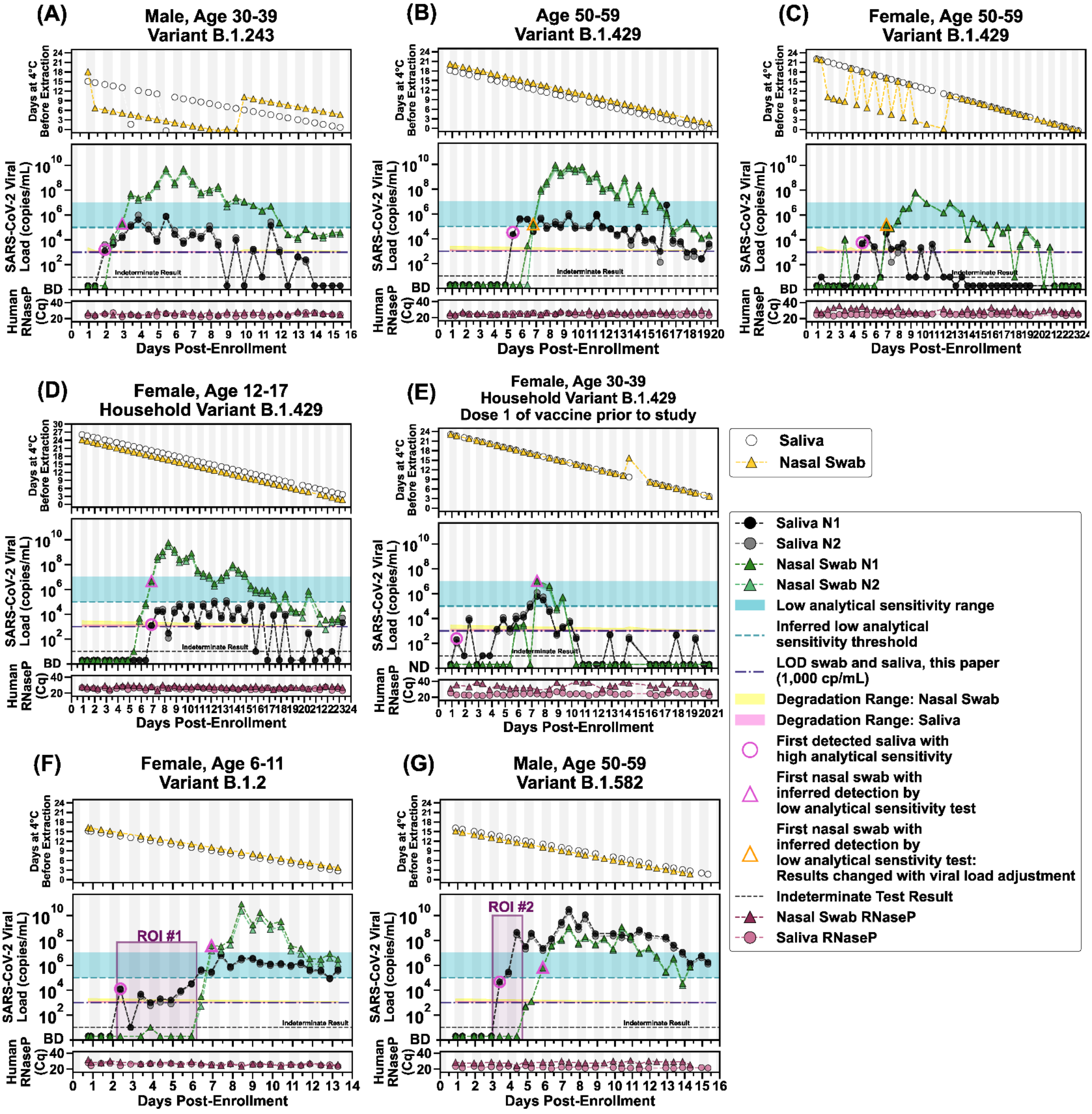

Figure S4. Predicted impact of SARS-CoV-2 RNA stability on quantitative viral loads shown in Fig. 2. (A-G) The time [days] of sample storage at 4 °C between sample collection and RNA extraction is shown in the topmost panels. Open circles represent saliva samples and yellow triangles represent the nasal swabs. Viral-load calculations are corrected for the median half-life (1 Cq decrease in RNA detected by RT-qPCR) of each sample type and the duration of storage at 4 °C before quantification (15 days for 2-fold decrease in detected RNA in nasal swabs and 51 days for 2-fold decreased in detected RNA in saliva). The degradation ranges, represented by a shaded yellow (nasal swab) or pink (saliva) region to represent how a measured value of 1,000 copies/mL may have degraded from concentrations in this range. As in Fig. 2, ND = not detected for Cqs ≥40 (see Methods for details). The limit of detection (LOD) of the saliva and nasal-swab assays used here (1,000 cp/mL) is indicated with the purple dashed line; low-analytical-sensitivity threshold

is indicated by the horizontal green dashed line; the low-analytical-sensitivity range (horizontal blue bar) is shown for reference. A diagnostic test does not provide reliable detection for samples with viral loads below its LOD. For each participant, the first detected saliva point is emphasized with a pink circle and their first nasal-swab point above the LOD of the ID NOW is emphasized with a pink triangle. Vertical shading in grey indicates nighttime (8pm – 8am). Internal control of *RNase P* gene Cqs from the CDC primer set are provided for each sample to compare self-sampling consistency and sample integrity (failed samples, where *RNase P* Cq ≥40, are not plotted). Samples with an indeterminate result by the CDC RT-qPCR assay are shown along the horizontal black dashed line. Participant gender, age range, and SARS-CoV-2 variant is given in each panel's title. Two regions of interest (ROI) are indicated by purple-shaded rectangles and discussed in the main text. For the two points that change interpretation with the viral-load adjustment, orange triangles show which new data points become the first nasal-swab point in range of low-analytical-sensitivity tests.

Table S1. Study participant demographic data. Figure 2 shows viral loads and symptoms data for the seven participants for whom we observed transmission during their enrollment in the study.

	**Age Range (Years)**	**Sex**	**Race; Ethnicity**	**Reported Medical Conditions Associated with Increased Risk of Severe COVID-19**[2]
Fig. 2A, Fig. S4A	30-39	Male	Other; Mexican/Mexican-American/Chicano (Salvadoran)	Diabetes
Fig. 2B, Fig. S4B	50-59	Male	Do not wish to respond; Mexican/Mexican-American/Chicano	None
Fig. 2C, Fig. S4C	50-59	Female	White; Mexican/Mexican-American/Chicano (Spanish-American from Spain)	None
Fig. 2D, Fig. S4D	12-17	Female	White; Mexican/Mexican-American/Chicano	None
Fig. 2E, Fig. S4E	30-39	Female	White; Mexican/Mexican-American/Chicano	None
Fig. 2F, Fig. S4F	6-11	Female	White; Non-Hispanic	None
Fig. 2G, Fig. S4G	50-59	Male	American Indian or Alaskan Native, White; Other Hispanic, Latinx or Spanish origin	Obesity

Supplementary Methods

Participant Population

This study is an extension of our previous study examining viral load in saliva.[3] Both studies were reviewed and approved by the Institutional Review Board of the California Institute of Technology, protocol #20-1026. All participants provided either written informed consent (or for minors ages 6-17, assent accompanied by parental permission), prior to enrollment. Household index cases were eligible for participation if they had recently (within 7 days) been diagnosed with COVID-19 by a CLIA laboratory test. Individuals were ineligible if they were hospitalized or if they were not fluent in either Spanish or English. All participant data were collected and managed using REDCap (Research Electronic Data Capture) on a server hosted at the California Institute of Technology. Demographic and health information for the seven participants can be found in Table S1.

Questionnaires and Symptom Monitoring

Acquisition of participant data was performed as described previously .[3] Briefly, upon enrollment each participant completed an online questionnaire regarding demographics, health factors, prior COVID-19 tests, COVID-19-like symptoms since February 2020, household infection-control practices, and perceptions of COVID-19 risk. Participants also filled out a post-study questionnaire in which they documented medications taken and their interactions with each household member during their enrollment.

Information on symptoms was collected twice daily in parallel with sample collection. Participants recorded any COVID-19-like symptoms (as defined by the CDC[4]) they were experiencing at the time of sample donation on a symptom-tracking card or on a custom app run on REDCap. Whenever possible, participants indicated the self-reported severity of each symptom. Participants were also given the opportunity to write-in additional symptoms or symptom details not otherwise listed.

Collection of Respiratory Specimens

Participants self-collected both their nasal-swab and saliva samples using the Spectrum SDNA-1000 Saliva Collection Kit (Spectrum Solutions LLC, Draper, UT, USA), which contains 1.5 mL of liquid buffer, at home twice per day (after waking up and before going to bed), per manufacturer guidelines. Of note, at the time of this writing, Spectrum devices are not approved for the collection of nasal-swab samples. Participants self-collected nasal-swab (1 swab) and saliva (~1.5 mL) samples in the Spectrum SDNA-1000 Saliva Collection Kit (Spectrum Solutions LLC, Draper, UT, USA), which contains 1.5mL of liquid buffer, at home twice per day (after waking up and before going to bed), per manufacturer's guidelines. Of note, at the time of this writing, Spectrum devices are not approved for the collection of nasal-swab samples.

Participants were instructed not to eat, drink, smoke, brush their teeth, use mouthwash, or chew gum for at least 30 min prior to donating. Prior to nasal-swab donation, participants were asked to gently blow their noses to remove debris. Participants were provided with one of the following types of sterile flocked swabs: Nest Oropharyngeal Specimen Collection Swabs (Cat. NST-202003, Stellar Scientific, Baltimore, MD, USA) Puritan HydraFlock Swab (Cat. 25-3000-H E30, Puritan, Guilford, ME, USA) or Copan USA FLOQSwab (Cat. 520CS01, VWR International, Radnor, PA, USA). Participants were instructed to swab each nostril for four complete rotations using the same swab while applying gentle pressure, then to break the tip of the swab into the Spectrum tube and securely screw on the cap. A parent or legal guardian assisted all minors with swab collection and they were instructed to wear a face covering during supervision. Tubes were labeled and packaged by the participants and transported at room temperature by a touch-free medical courier to the California Institute of Technology daily for analysis.

Upon receipt of the samples in the California Institute of Technology laboratory, each sample was inspected for quality. A sample failed quality control if the preservation buffer was not released from the Spectrum SDNA-1000 cap, or if sample tubes were leaking or otherwise unsafe to handle. Samples that failed quality control were not processed. Inactivated samples were stored at 4 °C and were equilibrated to room temperature before being processed with extraction protocols.

RNA Extraction Protocols

In initial testing, when combined with standard KingFisher MagMax sample-preparation protocols, these assays performed well to quantify heat-inactivated SARS-CoV-2 viral particles spiked into commercially available SARS-CoV-2 negative saliva and nasal fluid from pooled donors. However, the assay did not provide reliable quantification from freshly collected individual saliva samples with varying viscosity from positive participants in this study. Carryover of materials from some of the mucus-rich samples was inhibitory, as determined by RT-ddPCR analysis of dilutions of eluted RNA (data not shown). Following recommendations from ThermoFisher, the protocol was adjusted and described below. Briefly, we added a centrifugation step after proteinase k treatment to pellet the mucus-rich cell debris. We also include a third wash to improve RNA quality for viral genome sequencing. These steps reduced bead carryover into the eluate, as well as ddPCR inhibition.

Participant saliva and anterior-nares swab samples were extracted using the KingFisher Flex 96 instrument (ThermoFisher Scientific) with the MagMax Viral Pathogen I Nucleic Acid Isolation kit (Cat. A42352, Applied Biosystems, Waltham, MA, USA) guided by ThermoFisher technical notes for SARS-CoV-2 modification and saliva. Each extraction batch, depending on the sample type being extracted, contained a contrived SARS-CoV-2 negative control sample containing either 225 µL of Spectrum buffer mixed with 225 µL of commercial pooled human saliva (Lee Bio 991-05-P-PreC) or 240 µL of Spectrum buffer with 10 µL of pooled commercial nasal fluid (Lee Bio 991-13-P-PreC); a contrived SARS-CoV-2 positive control sample was also included in each extraction batch, with the formulations above, but with the Spectrum buffer spiked with 7,500 genomic copy equivalents/mL of heat-inactivated SARS-CoV-2 particles (BEI NR-52286).

Saliva and anterior-nares swab samples were prepared for purification by transferring 550 µl (for saliva) or 250 µl (for nasal swab) of each sample from its corresponding Spectrum buffer tube into a 1.5 mL lo-bind Eppendorf tube containing 10 µl (for saliva) or 5 µl (for nasal swab) of proteinase K. To maximize recovery of RNA off swabs, prior to transfer, pipet mixing was performed 5-7 times near the swab in the Spectrum tube before aliquoting into an Eppendorf tube. Saliva samples were vortexed for 30 sec in the Eppendorf tube. Samples were incubated at 65 °C for 10 min, then centrifuged at 13,000 x g for 1 min. Aliquots of 400 µl (for saliva) or 200 µl (for nasal swab) were transferred into a KingFisher 96 deep well plate (Cat. 95040450, ThermoFisher Scientific) and processed following KingFisher protocols MVP_400ul_3washes.bdz (for saliva) or MVP_200ul_2washes.bdz (for nasal swab). Ethanol washes were performed with 80% ethanol. Both sample types were eluted into 100 µl of MagMax viral pathogen elution buffer.

RT-qPCR

Quantification of SARS-CoV-2 was performed as previously described.[3] Briefly, the CDC[5] SARS-CoV-2 *N1* and *N2* gene primers and probes with an internal control targeting *RNase P* gene primer and probe were run in a multiplex RT-qPCR reaction using TaqPath 1-Step Rt-qPCR Mastermix (Cat. A15299, ThermoFisher Scientific). Reactions were run in duplicate on a CFX96 Real-Time Instrument (Bio-Rad Laboratories, Hercules, CA, USA).

RT-ddPCR

Reverse-transcription droplet digital PCR (RT-ddPCR) was performed using the Bio-Rad SARS-CoV-2 Droplet Digital PCR kit (Cat. 12013743, Bio-Rad). Swab samples were processed following the manufacturer's RUO protocol with 5.5 µl template per 22 µl reaction. A total of 42 participant nasal-swab samples were characterized by RT-ddPCR. Modifications were made for saliva samples by reducing the template addition to 2.75 µl per 22 µl reaction. A total of 63 participant saliva samples were characterized by RT-ddPCR. Prior to adding template, samples were diluted into digital range using nuclease-free water. Droplets were created using the QX200 Droplet Generator (Cat #1864002, Bio-Rad), thermocycling performed on Bio-Rad C1000 and detected using the QX200 Droplet Digital PCR system (Cat. 1864001, Bio-Rad). Samples were analyzed with QuantaSoft analysis Pro 1.0.595 software following Bio-Rad's RUO SARS-CoV-2 guidelines.[6]

Viral-load Calibration Curves

A calibration curve was prepared for both the saliva and nasal-swab protocols. Contrived samples were prepared with known concentrations (based on the certificate of analysis, COA) of heat-inactivated SARS-CoV-2 virus (3.75×10^8 GE/mL, Batch 70034991, Cat. NR-52286, BEI Resources, Manassas, VA, USA) in the inactivating buffer from the Spectrum SDNA-1000 Saliva Collection Kit (Spectrum Solutions LLC, Draper, UT, USA) and commercial, healthy human fluids were used as healthy human samples. Commercial pooled human saliva collected prior to November 2019 (Cat, 991-05-P, Lee Biosolutions, Maryland Heights, MO, USA) for the contrived saliva samples or commercial human nasal fluid collected prior to November 2019 (Cat No 991-13-P-PreC, Lee Biosolutions) for the contrived nasal-swab samples. Details of reagent volumes are described in the following two paragraphs for how the samples were prepared for both nasal swab and saliva calibration curves.

To establish the nasal-swab calibration curve (Fig. S2A), contrived samples were prepared by creating a dilution series of commercial heat-inactivated SARS-CoV-2 virus from BEI (3.75×10^8 GE/mL) in a 10-fold dilution series from 1×10^6 to 1×10^4 copies/mL with finer resolution down to our LOD at 1×10^3 copies/mL. Dilutions were prepared in Spectrum device inactivation buffer, to a volume of 768 µL, at concentrations of 0 copies/mL, 1,000 copies/mL, 2,500 copies/mL, 5,000 copies/mL, 7,500 copies/mL, 10,000 copies/mL, 100,000 copies/mL, and 1,000,000 copies/mL. To bring the volume to 800 µL total, 32 µL of healthy human nasal fluid collected prior to November 2019 (Cat No 991-13-P-PreC, Lee Biosolutions) was added. Triplicate extractions, 250 µL each, were performed according to the nasal-swab RNA extraction protocol (described above). Each extraction was run in triplicate RT-qPCR reactions.

To establish the saliva calibration curve (Fig. S2B), contrived samples were prepared by creating a dilution series of commercial BEI heat-inactivated SARS-CoV-2 virus in Spectrum device inactivation buffer at concentrations of 0 copies/mL, 1,000 copies/mL, 2,000 copies/mL, 4,000 copies/mL, 8,000 copies/mL, 16,000 copies/mL, 64,000 copies/mL, 256,000 copies/mL, and 1,020,000 copies/mL. Contrived samples were made by mixing 620 µL of each concentration of the dilution series with 620 µL of healthy pooled human saliva (Cat, 991-05-P, Lee Biosolutions). Triplicate extractions, 550 µL each, were performed according to the saliva RNA extraction protocol. Each extraction was run in triplicate RT-qPCR reactions.

Equations, calculated from the linear regression of the calibration curves, are shown below as Equations 1-4. These calibration curves are used to convert the Cq values obtained by RT-qPCR to viral load in each participant sample. For saliva, viral load is a calculation of viral copies/mL in the saliva corrected for dilution with the Spectrum buffer. We assumed that participants donate saliva to the fill line, matching the 1:1 dilution in Spectrum buffer recreated when preparing contrived samples for the saliva calibration curve. For nasal swabs, viral load is a calculation of the concentration of viral copies/mL released from the swab into the 1.5 mL of inactivating buffer (which is a similar volume as the 1-3 mL of viral transport media typically used for sample collection). Concentrations higher than 1,000,000 copies/mL could not be characterized due to a limitation of the available stock concentration of commercial inactivated SARS-CoV-2. To validate linear conversion was acceptable at

concentrations higher than 1,000,000 copies/mL, we compared RT-ddPCR and RT-qPCR quantification on some participant samples (Fig. 1) as described in the next section "Viral-load Quantification between qPCR and ddPCR assays."

$$(1)\ Saliva\ N1\ gene\ viral\ load\ [copies/mL] = 2^{((Cq-46.349)/-1.0357)}$$

$$(2)\ Saliva\ N2\ gene\ viral\ load\ [copies/mL] = 2^{((Cq-46.374)/-1.0759)}$$

$$(3)\ Nasal\ Swab\ N1\ gene\ viral\ load\ [copies/mL] = 2^{((Cq-48.221)/-1.0643)}$$

$$(4)\ Nasal\ Swab\ N2\ gene\ viral\ load\ [copies/mL] = 2^{((Cq-48.330)/-1.1044)}$$

Viral-load Quantification between qPCR and ddPCR Assays

Contrived saliva and nasal-swab calibration curve RT-qPCR data was converted into viral load (*N1* copies/mL) using Equations 1 and 3 listed in the above section. Calculated viral load was plotted against the theoretical input of heat-inactivated SARS-CoV-2.

Extending quantification capabilities above $1x10^6$ *N1* copies/mL was achieved using SARS-CoV-2-positive participant samples. Due to the limitation of the commercial SARS-CoV-2 standard concentration, we were not able to prepare contrived samples with SARS-CoV-2 input concentrations greater than $1x10^6$ copies/mL. To capture a range of participant samples over 7 orders of magnitude ($1x10^3$ to $1x10^{10}$ copies/mL SARS-CoV-2 *N1* gene), 63 saliva and 42 nasal-swab samples from SARS-CoV-2-positive participants were selected based on RT-qPCR data to quantify using RT-ddPCR. Using the geometric mean of the viral load computed from RT-qPCR and the calibration curves and the concentration measured by RT-ddPCR, we were able to evaluate the linearity of the calibration curve across the 7 orders of magnitude viral load seen in the participant samples (Fig. 1B-C). Samples were selected to capture the range of viral concentrations within our calibration curve and to the highest viral loads recorded for each sample type (nasal and saliva). The geometric means of RT-qPCR and RT-qPCR viral concentrations were calculated by taking the square root of RT-qPCR *N1* concentrations × RT-ddPCR *N1* concentration.

We observed excellent concordance between the calibration curve (Fig. 1A, complete data in Fig. S2), RT-qPCR and RT-ddPCR assays over the entire dynamic range of input concentrations (Fig. 1B-C), even though RT-qPCR eluents were run as-is and RT-ddPCR eluents from high-concentration samples were significantly diluted. For nasal-swab samples, RT-ddPCR values were slightly below the RT-qPCR values, however this difference was consistent across the entire dynamic range, indicating no concentration-dependent biases like enzymatic inhibition. We chose not to adjust the calibration curve to fit RT-ddPCR values; we reported the concentrations based on the calibration curves derived from the certificate of analysis from the BEI Resources reference material. For saliva samples, all points tightly clustered around the x=y line (Fig. 1 A-C).

Establishment of Limit of Detection

Results of the calibration curve (Fig. S2 A,B) demonstrated 3 of 3 replicates detected at 1,000 copies/mL saliva (for saliva) and 1,000 copies/mL buffer (for nasal swabs). For each sample type (saliva, nasal swab), 20 contrived samples with the equivalent of 1,000 copies/mL were prepared as described above, individually extracted as described above, and subjected to RT-qPCR as described above. The LOD for each sample type through the

workflow was considered established if a positive result for detection (as defined in the EUA for the CDC RT-qPCR assay) was obtained for ≥ 19 of 20 (≥95% as required by FDA EUA guidelines for determining LOD) of replicates at the input concentration (Fig. S1 A,B).

Three of three replicate sample extractions included in the calibration curves for both contrived nasal-swab samples and contrived saliva samples spiked with heat-inactivated SARS-CoV-2 particles at a concentration of 1,000 copies/mL were detected by RT-qPCR, prompting testing of additional 20 replicates of each sample type spiked at that concentration, individually extracted, and tested by RT-qPCR to establishment of the LOD for our RT-qPCR assay. For both sample types (saliva and nasal swabs), 20 of 20 replicates were positive for SARS-CoV-2 (Fig. S1 A,B), establishing 1,000 copies/mL of saliva and 1,000 copies/mL of swab buffer as the high-sensitivity LOD for our RT-qPCR assays.

Threshold to Infer Performance of Tests with Low Analytical Sensitivity

The threshold of $1.0\text{x}10^5$ copies/mL is applied generally to both saliva and nasal swabs viral loads (copies/mL) to infer detection by a test with low analytical sensitivity. The rationale to use this threshold is to demonstrate a best-case scenario performance of tests with low-analytical-sensitivity (from the low-analytical-sensitivity range $1.0\text{x}10^5 – 1.0\text{x}10^7$ copies/mL used in this paper). The comparisons in the paper would be more dramatic if a low-analytical-sensitivity threshold greater than this number was selected.

Data Analysis

Before we converted Cq values to viral load, we used Cq cutoffs based on the CDC guidelines[5] to define samples as positive, negative, indeterminate, or invalid (fail), and then excluded from the viral-load plots any points that failed, and any samples whose RNase P Cq values ≥40. Because we ran duplicate RT-qPCR reactions, the mean Cq of positive reactions was used for conversion to viral load.

Figure 3A percentages are calculated by Equation 5, where the percentage positive by a test of a given analytical sensitivity (high-analytical-sensitivity results are all measured values, by our internal test with an LOD ≤ 1000 cp/mL; low-analytical-sensitivity results ,are measured values at or above a threshold of $1.0\text{x}10^5$ cp/mL):

$$(5)\ Percent\ Positive_{as} = \frac{n}{N} \times 100$$

Where “as” refers to the analytical sensitivity. In Equation 5, “N” is defined as the total number of participants with saliva samples passing quality-control evaluations (see Methods) for safety and Human *RNaseP* gene Cq threshold at the corresponding aligned time point in column “Days from First Positive Results in Either Sample Type.” Maximum denominator of number of 7, corresponding to the number of participants in the study and each participant has a maximum of one sample per time point. Numbers may vary before day 0 as each participant had a variable number of negative test results before first detected SARS-CoV-2 RNA. In Equation 5, “n” represents the number of participants, at a given time point, with detectable SARS-CoV-2 RNA (see RT-qPCR methods) in the sample type (saliva or nasal swab) using a high-analytical-sensitivity assay. For predicting performance of each sample type (saliva or nasal swab) with a test of low analytical sensitivity, “n” is defined as the number of participants with a SARS-CoV-2 *N1* gene viral load of SARS-CoV-2 greater than $1.0\text{x}10^5$ copies/mL (cp/mL) in samples, which would indicate an inferred positive result using a low-sensitivity assay with an LOD of SARS-CoV-2 *N1* gene viral load of $1.0\text{x}10^5$ copies/mL. Details of the calculations are included in the Data_Annotation file on CaltechDATA.

Figure 3D considers only samples collected within 10 days after the assigned first positive result were analyzed to consider symptoms relevant to an early infection. The first date of positive result observed using our high-analytical-sensitivity assay (either sample type) was assigned for each participant shown in the panels of Figure 2 and days 0-10 were analyzed for panel D.

Samples were designated as being collected while symptomatic if the participant reported experiencing one or more COVID-19-like symptoms at the time of sample collection; if no COVID-19 like symptoms were reported, the sample was designated as "No Symptoms Reported." Samples were defined as either positive, negative, or indeterminate by our high-analytical-sensitivity assay, based on the criteria from the manufacturer of the RT-qPCR assay, detailed above. Samples were inferred as either positive or negative by a low-analytical-sensitivity assay if the viral load measured in the sample was greater than our inferred low-analytical-sensitivity threshold, $1.0x10^5$ copies/mL.

Figure 3D utilizes Equation 5, where "N" is defined as the number of participant samples positive for SARS-CoV-2 RNA within the symptomatic categories defined in the first 10 days of detectable infection (criteria above). There were 97 saliva and 95 nasal-swab samples collected while symptomatic, and 46 saliva and 44 nasal-swab samples collected with the participant reporting no symptoms. The value of "n" corresponds to the percent positive by either observed positivity by our high-analytical-sensitivity assay or inferred positive by a low-analytical-sensitivity assay as the numerator over the denominator corresponding to that sample type and symptom status, multiplied by 100%.

RNAseq

Saliva and nasal-swab samples below *N1* Cq of 26 were sent to Chan Zuckerberg Biohub for SARS-CoV-2 viral genome sequencing, a modification of Deng et al. (2020)[7] as described in Gorzynski et al. (2020).[8] Sequences were assigned pangolin lineages described by Rambaut *et al*. (2020)[9] using Phylogenetic Assignment of Named Global outbreak LINeages software v2.3.2 (github.com/cov-lineages/pangolin). Consequences viral genomes were submitted to GISAID by Chan Zuckerberg Biohub, see data availability section for accession id details.

SARS-CoV-2 RNA Stability at 4 °C

As described above, each extraction batch included a contrived sample spiked with SARS-CoV-2 heat-inactivated particles. For all available saliva or nasal-swab extraction batches, the Cq value of the SARS-CoV-2 *N1* gene in the contrived SARS-CoV-2 positive extraction control was collected. The standard deviation of these measurements was calculated and used to establish a threshold for expected noise between repeat extractions of the same sample. To assess samples for evidence of SARS-CoV-2 RNA degradation, any participant sample that had more than one extraction replicate performed were analyzed. Samples where the difference in Cq values between the extractions was less than the threshold of expected noise between replicate extractions were defined as degradation not observed, (DNO). For samples where the difference was above this threshold, the time for 1 Cq increase (2-fold decrease) in RNA detected by RT-qPCR is described by the term half-life, which was calculated according to Equation 6, below:

$$(6)\ t_{1/2} = \frac{-ln2}{k}$$

Where "k" is defined as the slope of the linear regression of the natural logarithm of the viral load vs. extraction date (relative to sample collection date). The median over the entire dataset (saliva or swab) was used as a point estimate of RNA half-life. The median point was determined to be 15.0 days for nasal swabs and 51.0 days for saliva.

Calculations that predict the impact of storage time at 4 °C and RNA stability on viral load are calculated according to Equation 7, below.

$$(7)\ y_{adj} = y_{deg} 2^{\frac{\Delta t}{t_{1/2}}}$$

Where y_{adj} is defined as the adjusted viral load, y_{deg} is defined as the viral load before adjustment for degradation (as calculated by Equations 1-4), and $t_{1/2}$ is defined as the RNA half-life, shown in Equation 5.

All samples were stored at 4 °C before extraction; time of storage varied between 0-27 days. The stability of SARS-CoV-2 RNA in nasal-swab samples was slightly lower (1 Cq loss of RNA detected after a median of 15 days) than the stability of SARS-CoV-2 RNA in saliva samples (1 Cq loss of RNA detected after a median of 51 days) (Fig. S3). An assessment of how viral-load measurements in Fig. 2 may have been affected by time between sample collection and quantification is included in Fig. S4. Given the large dynamic range of the viral loads in these samples (~24 Cq or about 10,000,000 fold), we considered stability corresponding to a 1 Cq (2 fold) loss to be adequate.

The predicted impact of RNA degradation on the comparisons of high-analytical-sensitivity saliva to inferred low-analytical-sensitivity nasal testing is shown in Fig. S4. Accounting for potential decreases of viral RNA in the nasal swab resulting from delays between sample collection and quantification only impact the interpretation of two points, conservatively decreasing the delay from 2.0 to 1.5 day for the first participant (Fig. 2B and Fig. S4B) and from 3.0 to 2.0 days for the third participant (Fig. 2C and Fig. S4C).

Supplementary Discussion

Three participants (Fig. 1C–E) were infected with the same variant, B.1.429 (CAL20), classified as a variant-of-concern at the time of this study. The SARS-CoV-2 variant for the participants in Fig. 1D and Fig. 1E were inferred from the sequenced sample of the household's presumed index case. Saliva viral loads for each of these participants (Fig. 2C-E) were low. Of note, the participants in Fig. 2C and 2E showed high *RNase P* Cq values (indicating low concentration of the human control target); and variability of *RNase P* Cq values across the nasal-swab samples suggests that inconsistent swab-sampling quality could have impacted these participants' viral-load data and should be taken into account when interpreting those data.

Beyond outbreak prevention and control, early detection of COVID-19 may also be useful for individual patient care, as high-risk patients who are identified early can be monitored and have treatment initiated swiftly if it becomes appropriate. Several treatments show exclusive or increased efficacy only when given early in the infection. The advantage of earlier treatment initiation is likely due to reduction of viral replication either directly or by promotion of an early effective immune response, which prevents a later exaggerated inflammatory response.[10] Results of the ACTT-1 trial demonstrated a survival benefit in patients for whom Remdesivir was initiated in the early stages of treatment (supplemental oxygen only), but that benefit was lost once disease progressed, and advanced respiratory support was needed.[10-12] More recently, the MOVe-OUT clinical trial demonstrated the efficacy of molnupiravir when (per trial inclusion criteria) initiated among outpatients within the first five days from symptom onset, whereas the inpatient study (MOVe-IN) did not proceed to Phase 3, as clinical benefit was not observed for hospitalized patients with a longer duration of symptoms prior to initiation of the treatment.[10] Other therapies, such as plasma and monoclonal antibody therapies (bamlanivimab or casirivimab plus imdevimab) show similar clinical benefits in early initiation of treatment.[11-16]

Although national vaccination efforts are reducing severe COVID-19 outcomes in the U.S., a sizable portion of the world's population is likely to remain unvaccinated due to limited vaccine availability, medical ineligibility (in the U.S., children under 5 years of age are not yet eligible), or personal preference. Thus, testing remains an important tool for preventing outbreaks among children in schools and daycare facilities (where children under age 2 generally do not wear masks), which may spread to the community and increase rates of infection among high-risk and unvaccinated individuals. Tests that detect early infections are also important to prevent viral transmission in congregate settings with high-risk or unvaccinated populations, such as hospitals, college dormitories, homeless shelters, correctional facilities, summer camps for children, elementary schools, and long-term care facilities.

As new SARS-CoV-2 variants emerge, quantitative studies of the kinetics of early-stage viral loads must be continually updated in follow-up studies. Importantly, such studies should be undertaken in people of a wide range of ethnicities, races, health conditions, vaccination status, and ages. Breakthrough cases are often asymptomatic[17] and recent evidence suggests that vaccinated individuals may transmit infections from the new variants, including Delta.[18] Another reason for continued monitoring of early viral kinetics is that viral evolution, including of host tropism, can markedly diminish the effectiveness of a diagnostic strategy. In one study, decreased clinical sensitivity of NP swabs was observed in SARS-CoV-2 variant B.1.616,[19] which may indicate a tropism shift of the virus into lower-respiratory compartments. Finally, quantitative data must be acquired in parallel with viral-culture data to understand the viral loads and phases of infection that are most relevant to transmission.

Early detection of infection clearly reduces community transmission, however for most of the COVID-19 pandemic, policy makers have had to develop testing strategies in the absence of quantitative data on viral kinetics from the earliest stage of infection. Lacking such data-based guidance, diagnostic tests have been used incorrectly (with false-negative results due to using tests with insufficient sensitivity) in several scenarios, resulting in outbreaks that could have been prevented with an appropriate testing strategy.[20-26]

One barrier to implementing such more advanced testing strategies is availability of appropriate tests. Because the optimal sample type for early detection might be different for different populations, or might change as new variants emerge, tests with robust high analytical sensitivity across multiple sample types are needed. Developing such tests is challenging because it requires incorporating robust sample-preparation technology to purify and concentrate pathogen nucleic acids from diverse human matrices, from upper respiratory (e.g. fluids from the nasal, nasopharyngeal, oral and oropharyngeal compartments, captured in swabs or saliva) to lower respiratory samples (e.g. sputum, tracheal aspirate, bronchoalveolar lavage). It is even more challenging to incorporate such sample-preparation technology into tests that can be broadly deployed—at very low cost—at the point of care in limited-resource settings (such as schools, homes, and businesses, and especially in the developing countries). Development of such highly sensitive, rapid, and inexpensive tests with broad sample-type compatibility is urgently needed.

Supplemental References

1 CDC. Real-Time RT-PCR Panel for Detection 2019-Novel Coronavirus. (Centers for Disease Control and Prevention, Respiratory Viruses Branch, Division of Viral Diseases, 2020).

2 CDC. *COVID-19: People with Certain Medical Conditions*, <https://www.cdc.gov/coronavirus/2019-ncov/need-extra-precautions/people-with-medical-conditions.html> (2021).

3 Winnett, A. *et al.* SARS-CoV-2 Viral Load in Saliva Rises Gradually and to Moderate Levels in Some Humans. *medRxiv*, 2020.2012.2009.20239467, doi:10.1101/2020.12.09.20239467 (2020).

4 CDC. Symptoms of Coronavirus. https://www.cdc.gov/coronavirus/2019-ncov/symptoms-testing/symptoms.html (2020).

5 FDA. *CDC 2019-Novel Coronavirus (2019-nCoV) Real-Time RT-PCR Diagnostic Panel (EUA)*, <https://www.fda.gov/media/134922/download> (2020).

6 Bio-Rad. *Bio-Rad SARS-CoV-2 ddPCR Kit, Instructions for Use*, <https://www.bio-rad.com/webroot/web/pdf/lsr/literature/10000130776.pdf> (2020).

7 Deng, X. *et al.* Metagenomic sequencing with spiked primer enrichment for viral diagnostics and genomic surveillance. *Nature Microbiology* **5**, 443-454, doi:10.1038/s41564-019-0637-9 (2020).

8 Gorzynski, J. E. *et al.* High-throughput SARS-CoV-2 and host genome sequencing from single nasopharyngeal swabs. *medRxiv*, 2020.2007.2027.20163147, doi:10.1101/2020.07.27.20163147 (2020).

9 Rambaut, A. *et al.* A dynamic nomenclature proposal for SARS-CoV-2 lineages to assist genomic epidemiology. *Nature Microbiology* **5**, 1403-1407, doi:10.1038/s41564-020-0770-5 (2020).

10 Merck & Co. Inc. *Merck and Ridgeback Biotherapeutics Provide Update on Progress of Clinical Development Program for Molnupiravir, an Investigational Oral Therapeutic for the Treatment of Mild-to-Moderate COVID-19*, <https://www.merck.com/news/merck-and-ridgeback-biotherapeutics-provide-update-on-progress-of-clinical-development-program-for-molnupiravir-an-investigational-oral-therapeutic-for-the-treatment-of-mild-to-moderate-covid-19/> (2021).

11 Simonovich, V. A. *et al.* A Randomized Trial of Convalescent Plasma in Covid-19 Severe Pneumonia. *N. Engl. J. Med.* **384**, 619-629, doi:10.1056/NEJMoa2031304 (2020).

12 Libster, R. *et al.* Prevention of severe COVID-19 in the elderly by early high-titer plasma. *medRxiv*, 2020.2011.2020.20234013, doi:10.1101/2020.11.20.20234013 (2020).

13 Lundgren, J. D. *et al.* A Neutralizing Monoclonal Antibody for Hospitalized Patients with Covid-19. *N. Engl. J. Med.* **384**, 905-914, doi:10.1056/NEJMoa2033130 (2021).

14 Chen, P. *et al.* SARS-CoV-2 Neutralizing Antibody LY-CoV555 in Outpatients with Covid-19. *N. Engl. J. Med.* **384**, 229-237, doi:10.1056/NEJMoa2029849 (2021).

15 Weinreich, D. M. *et al.* REGN-COV2, a Neutralizing Antibody Cocktail, in Outpatients with Covid-19. *N. Engl. J. Med.* **384**, 238-251, doi:10.1056/NEJMoa2035002 (2021).

16 FDA. *Fact sheet for health care providers emergency use authorization (EUA) of casirivimab and imdevimab*, <https://www.fda.gov/media/143892/download> (2020).

17 CDC. COVID-19 Vaccine Breakthrough Infections Reported to CDC — United States, January 1–April 30, 2021. *MMR Morb Mortal Wkly Rep* **70**, 792-793 (2021).

18 Brown, C., Vostok, J., Johnson, H. & Et al. Outbreak of SARS-CoV-2 Infections, Including COVID-19 Vaccine Breakthrough Infections, Associated with Large Public Gatherings — Barnstable County, Massachusetts, July 2021. *MMWR Morb Mortal Wkly Rep* **70**, 1059-1062 (2021).

19 Fillatre, P. *et al.* A new SARS-CoV-2 variant poorly detected by RT-PCR on nasopharyngeal samples, with high lethality. *medRxiv*, 2021.2005.2005.21256690, doi:10.1101/2021.05.05.21256690 (2021).

20 Pray IW, Gibbons-Burgener SN, Rosenberg AZ & et al. COVID-19 Outbreak at an Overnight Summer School Retreat — Wisconsin, July–August 2020. *MMR Morb Mortal Wkly Rep* **69**, 1600-1604 (2020).

21 Moreno, G. K. *et al.* Severe Acute Respiratory Syndrome Coronavirus 2 Transmission in Intercollegiate Athletics Not Fully Mitigated With Daily Antigen Testing. *Clin. Infect. Dis.* **73**, S45-S53, doi:10.1093/cid/ciab343 (2021).

22 Winichakoon, P. *et al.* Negative Nasopharyngeal and Oropharyngeal Swabs Do Not Rule Out COVID-19. *J. Clin. Microbiol.* **58**, e00297-00220, doi:10.1128/JCM.00297-20 (2020).

23 Schwartz, N. G. *et al.* Adolescent with COVID-19 as the Source of an Outbreak at a 3-Week Family Gathering - Four States, June-July 2020. *MMWR. Morbidity and mortality weekly report* **69**, 1457-1459, doi:10.15585/mmwr.mm6940e2 (2020).

24 Cao, G. *et al.* The Potential Transmission of SARS-CoV-2 from Patients with Negative RT-PCR Swab Tests to Others: Two Related Clusters of COVID-19 Outbreak. *Jpn. J. Infect. Dis.* **73**, 399-403, doi:10.7883/yoken.JJID.2020.165 (2020).

25 Hagan, L. M. *et al.* Mass Testing for SARS-CoV-2 in 16 Prisons and Jails - Six Jurisdictions, United States, April-May 2020. *MMWR. Morbidity and mortality weekly report* **69**, 1139-1143, doi:10.15585/mmwr.mm6933a3 (2020).

26 Szablewski, C. M. *et al.* SARS-CoV-2 Transmission and Infection Among Attendees of an Overnight Camp - Georgia, June 2020. *MMWR. Morbidity and mortality weekly report* **69**, 1023-1025, doi:10.15585/mmwr.mm6931e1 (2020).

Author Contributions

Listed alphabetically by last name

Reid Akana (RA): Assisted in literature analysis with ES, MKP, AVW, MC; collaborated with AVW in creating digital participant symptom surveys; assisted with data quality control/curation with JJ, NWS, NS; collaborated with ES, JJ to write data analysis/visualization code; created current laboratory information management system (LIMS) for specimen logging and tracking. Creation of iOS application for sample logging/tracking. Configured a SQL database for data storage. Created an Apache server and websites to view study data. Configured FTPS server to catalog PCR data. Wrote a Python package to access study data. Worked with ES, AVW, AR to implement logic that prioritized specimen extraction order. Collaborated with ES, MKP, AVW, AR in analyzing RNA stability. Created supplementary figures 3B and 3C.

Jacob T. Barlow (JTB): Created initial specimen tracking database to aid in specimen logging and tracking. Maintenance of database and implementation of corrections. Feedback on manuscript draft.

Alyssa M. Carter (AMC): Received and logged specimens, and performed sample QC. Prepared reagents for and assisted with RNA extractions. Performed RT-qPCR and analyzed RT-qPCR data for both time series and screening experiments. Performed some of the initial experiments that assessed RNA stability in nasal swab samples

Matthew M. Cooper (MMC): Collaborated with AVW, MF, NS, YG, RFI, on study design and recruitment strategies. Co-wrote initial IRB protocol and informed consent with AVW and NS; assisted in the writing of the enrollment questionnaire; developed laboratory sample processing workflow for saliva with AVW and AER; performed sample processing on subset of samples; funding acquisition; collaborated with AR to write data processing/visualization code for observing household transmission events for active study participants. Contributor to the design of the calibration curve for saliva LOD experiments.

Matthew Feaster (MF): Co-investigator; collaborated with AVW, MMC, NS, YG, RFI on study design and recruitment strategies; provided guidance and expertise on SARS-CoV-2 epidemiology and local trends.

Ying-Ying Goh (YYG): Co-investigator; collaborated with AVW, MMC, NS, MF, RFI on study design and recruitment strategies; provided guidance and expertise on SARS-CoV-2 epidemiology and local trends.

Rustem F. Ismagilov (RFI): Co-investigator; collaborated with AVW, MMC, NS, MF, YYG on study design and recruitment strategies; provided leadership, technical guidance, oversight, and was responsible for obtaining funding for the study.

Jenny Ji (JJ): Contributed to study design and study organization and implementation with NS and JAR; co-wrote enrollment questionnaire with NS and AVW. Major contributor to curation of participant symptom data. Provided quality control of participant data with RA, NS, NWS. Major contributor to the symptom data analysis and visualization shown in Fig. 2.

Michael K. Porter (MKP): Performed specimen logging and QC, RNA extractions, RT-qPCR, data processing. Performed data acquisition and analysis for and made Figure S1 with AVW. Prepared participant sample collection materials and helped with supplies acquisition. Assisted in literature analysis with ES, RA, AVW.

Jessica A. Reyes (JAR): Study coordinator; collaborated with NS, AVW, NWS, and RFI on recruitment strategies, translated study materials into Spanish, co-wrote informational sheets with AVW and NS; created instructional videos for participants; enrolled and maintained study participants with NS and NWS.

Anna E. Romano (AER): Developed laboratory swab sample processing workflow with ES. Optimized extraction and ddPCR protocols working with vendor scientists. Created budgets and managed, planned, and purchased

reagents and supplies; developed and validated method for RT-qPCR and RT-digital droplet PCR analysis for saliva & swab samples with MMC, and AVW. Performed specimen logging and QC, RNA extractions, RT-qPCR and RT-digital droplet PCR; Design of saliva calibration curve experiment. Analyzed ddPCR data for participant and calibration curve data included in Figure 1. Interpretation of sequence data with AVW. Prepared Figure 1 and SI Figure S2 with ES and AVW; Collaborated with ES and RA to generate and curate data for RNA stability analysis. Managing logistics for the expansion of the BSL-2+ lab space with ESS. Edited manuscript.

Emily S. Savela (ESS): Coordinated the laboratory team and division of lab work, coordinated lab schedules to ensure completion of time-sensitive analyses of participant samples while complying with COVID-19 lab occupancy restrictions and biosafety requirements. Performed initial nasal-swab workflow validation experiments with AER. Major contributor to workflow validation, methods, biosafety SOPs, and sample storage. Developed a plan for, and executed, the long term sample storage for efficient, safe, storage. Performed specimen logging and QC, RNA extractions, RT-qPCR, data processing, and conducted biosafety training. Performed the data curation and data analysis for Figure 2. Made Figure 2. Minor contributor to symptoms data analysis and visualization with JJ and RA for Figure 2. Experimental design and RNA extractions of the samples, to Figure 1A and minor contributor to Figure S2A with AER. Managing logistics for the expansion of the BSL-2+ lab space with AER and biohazardous waste pickups. Collaborated with ES, RA, and AVW to generate data for and curated data set to assess viral RNA stability (Fig. S3). Prepared Fig. S4. Co-wrote the manuscript. Verified the underlying data with AVW.

Noah W. Schlenker (NWS): Study coordinator; collaborated with NS, AVW, JAR, and RFI on recruitment strategies; enrolled and maintained study participants with NS and JAR; study-data quality control, curation and archiving with RA, JJ, and NS.

Natasha Shelby (NS): Study administrator; collaborated with AVW, MMC, RFI, YG, MF on initial study design and recruitment strategies; co-wrote IRB protocol and informed consent with AVW and MMC; co-wrote enrollment questionnaire with AVW and JJ; co-wrote participant informational sheets with AVW and JAR; enrolled and maintained study participants with JAR and NWS; study-data quality control, curation and archiving with RA, JJ, and NWS; reagents and supplies acquisition; assembled Table S1; managed citations and reference library; co-wrote and edited the manuscript.

Colten Tognazzini (CT): Coordinated the recruitment efforts at PPHD with case investigators and contact tracers; provided guidance and expertise on SARS-CoV-2 epidemiology and local trends.

Alexander Viloria Winnett (AVW): Collaborated with MMC, NS, RFI, YG, MF on initial study design and recruitment strategies; co-wrote IRB protocol and informed consent with MMC and NS; co-wrote enrollment questionnaire with NS and JJ; co-wrote participant informational sheets with NS and JAR and digital survey; developed and validated methods for saliva and nasal-swab sample collection; developed and validated methods for RT-qPCR and RT-digital droplet PCR analysis for saliva and swab samples with AER, ESS, MMC; reagents and supplies acquisition; funding acquisition; developed laboratory sample processing workflow with AER, ESS, and MMC; performed specimen logging and QC, nucleic acid extraction, RT-qPCR, data processing – including experimental data generation for saliva calibration curve (Fig. 1, Fig. S2) designed with MMC and AER, establishment of nasal swab limit of detection (Fig. S1), and viral load timeseries data (Fig. 2) with ESS, AER, MKP, and AMC; interpreted sequencing data with AER; analyzed viral load timeseries data to visualize trends (Fig. 3) with ESS; generated, analyzed and visualized data to assess degradation of viral RNA in saliva and nasal swab samples (Fig. S3) with RA, ESS, and AER; literature analysis with RA, ESS, and MKP; co-wrote sections of the manuscript outlined by ESS and RFI, edited the manuscript. Verified the underlying data with ESS.
